# The Interaction of Aging and Cellular Stress Contributes to Pathogenesis in Mouse and Human Huntington Disease Neurons

**DOI:** 10.3389/fnagi.2020.524369

**Published:** 2020-09-18

**Authors:** Emily Machiela, Ritika Jeloka, Nicholas S. Caron, Shagun Mehta, Mandi E. Schmidt, Helen J. E. Baddeley, Colton M. Tom, Nalini Polturi, Yuanyun Xie, Virginia B. Mattis, Michael R. Hayden, Amber L. Southwell

**Affiliations:** ^1^Burnett School of Biomedical Sciences, University of Central Florida, Orlando, FL, United States; ^2^Centre for Molecular Medicine and Therapeutics, University of British Columbia, Vancouver, BC, Canada; ^3^The Board of Governors Regenerative Medicine Institute, Cedars-Sinai Medical Center, Los Angeles, CA, United States

**Keywords:** Huntington (disease), iPSC (induced pluripotent stem cell), primary neuron culture, mouse models of disease, neurodegenaration, DNA damage

## Abstract

Huntington disease (HD) is a fatal, inherited neurodegenerative disorder caused by a mutation in the huntingtin (*HTT*) gene. While mutant HTT is present ubiquitously throughout life, HD onset typically occurs in mid-life. Oxidative damage accumulates in the aging brain and is a feature of HD. We sought to interrogate the roles and interaction of age and oxidative stress in HD using primary Hu97/18 mouse neurons, neurons differentiated from HD patient induced pluripotent stem cells (iPSCs), and the brains of HD mice. We find that primary neurons must be matured in culture for canonical stress responses to occur. Furthermore, when aging is accelerated in mature HD neurons, mutant HTT accumulates and sensitivity to oxidative stress is selectively enhanced. Furthermore, we observe HD-specific phenotypes in neurons and mouse brains that have undergone accelerated aging, including a selective increase in DNA damage. These findings suggest a role for aging in HD pathogenesis and an interaction between the biological age of HD neurons and sensitivity to exogenous stress.

## Introduction

Huntington disease (HD) is an autosomal dominant neurodegenerative disease caused by an expanded polyglutamine encoding CAG tract in the huntingtin (*HTT*) gene (MacDonald et al., 1993). HD neuropathology originates with degeneration of striatal medium spiny neurons (MSNs), followed by white matter loss and widespread forebrain atrophy. HD is characterized by progressive movement, psychiatric, cognitive, and behavioral abnormalities, with motor symptoms typically beginning between ages 35 and 45 (Bates et al., 2015), and death occurring 10–20 years after symptom onset. Currently, there are no disease-modifying therapies for HD.

Age-of-onset of HD is negatively correlated with CAG tract length (Andrew et al., 1993a,b; Snell et al., 1993). However, this only explains ~50–70% of the variation in disease age-of-onset, with other genetic and environmental factors making up the remainder (Wexler et al., 2004). Patients with the same expanded CAG tract length can have differences in disease onset of up to 50 years. Additionally, those with identical mutations can progress at different rates. This suggests that factors aside from the CAG repeat can have a strong influence on disease pathogenesis.

A genome-wide association study (GWAS) conducted in HD patients has identified single nucleotide polymorphisms (SNPs) in genes associated with early and late disease onset (Genetic Modifiers of Huntington’s Disease, 2015). Several loci that impact disease onset were identified. Pathway analysis of these loci revealed the strongest signals were found in genes involved in DNA damage repair. Specifically, these genes are responsible for pathways that repair breaks in DNA associated with damage from stressors, such as oxidative stress. More recently, a GWAS was performed to identify factors that influence the progression of HD. Similar to findings for the age of onset, the SNPs with the strongest signals identified to influence the progression of HD were found in genes recruited to DNA after oxidative DNA damage, for base-excision repair (Lai et al., 2016; Moss et al., 2017).

In HD, there is evidence for both reduced oxidative stress responses and increased oxidative damage in neurons (Kovtun et al., 2007; Johri and Beal, 2012; Rotblat et al., 2014; Sepers and Raymond, 2014). Reactive oxygen species (ROS) are by-products of normal cellular metabolism, which are cleared primarily by endogenous antioxidant systems in the cell (Beckhauser et al., 2016). Oxidative stress occurs when oxidants overwhelm antioxidants, which leads to oxidative damage to proteins, nucleic acids, and other biomolecules. Increased markers of oxidative damage such as protein carbonylation, lipid peroxidation, and DNA damage have been found in postmortem HD brains and the brains of HD mice (Browne et al., 1999; Bogdanov et al., 2001; Browne and Beal, 2006). Additionally, in the blood of HD patients, decreased antioxidant protein concentrations and increased oxidative damage have been found (Chen et al., 2007; Klepac et al., 2007), suggesting that the impaired oxidative stress response occurs in both the brain and peripheral tissues of HD patients. The consequences of oxidative damage include loss of protein or organelle function, and permanent damage or failed repair of DNA and RNA. Oxidative damage can also increase the somatic expansion of the CAG repeat tract in HD neurons, a phenomenon associated with earlier HD onset (Kovtun et al., 2007; Jonson et al., 2013). While a small percentage of HD patients experience the juvenile onset form of the disease (Nance and Myers, 2001), HD is primarily a disease of aging; patients live with the mutation for many decades before symptom onset occurs. Many of the hallmarks of aging (López-Otín et al., 2013) are also hallmarks of HD cellular pathogenesis, including mitochondrial dysfunction leading to ROS and genomic instability from oxidative DNA damage. Combined with evidence of advanced biological age in HD brains (Horvath et al., 2016; Grima et al., 2017), age-related defects in the nuclear pore complex of HD neurons (Grima et al., 2017), and the fact that aging can render MSNs vulnerable to mutant HTT (mtHTT) toxicity (Diguet et al., 2009) suggests that aging may play an active role in HD pathogenesis.

In this work, we sought to better understand the relationship between age, cellular stress, and pathogenesis of HD to identify potential mechanisms for delaying the onset or slowing the progression of the disease. We investigated differences in the oxidative stress response in immature, mature, and aged HD and control neurons. One difficultly in modeling late-onset diseases such as HD is the lack of aging markers and lack of aging phenotypes in typical model systems. Rodents have relatively short lifespans, and most studies are performed in young animals for time and cost-efficiency. Moreover, both rodent primary neurons and neurons derived from patient induced pluripotent stem cells (iPSCs) are developmentally reset, and therefore embryonic-like in nature. Embryonic neurons from adult-onset HD patients are robust with few overt phenotypes (The HD iPSC Consortium, 2012; Mattis et al., 2015; Studer et al., 2015), thus not fully recapitulating the cellular pathogenesis of HD. To overcome this, we induced aging in neurons using progerin. Progerin is a truncated form of the lamin A protein that causes Hutchinson-Gilford Progeria Syndrome, a disease of premature aging (Eriksson et al., 2003). Progerin expression causes cellular senescence in normal fibroblasts, and accumulation of progerin occurs during normal aging (McClintock et al., 2007; Cao et al., 2011). While lamin A expression is low in the brain (Jung et al., 2013), progerin expression has previously been shown to induce aging and uncover aging-related phenotypes in iPSC-derived neurons from Parkinson disease patients (Miller et al., 2013).

We investigated both basal levels of oxidative damage and the response to exogenous oxidative stress in immature and mature primary forebrain neurons from HD mice. Additionally, we investigated if inducing aging *in vitro* in HD primary cortical neurons as well as HD patient iPSC-derived neurons and *in vivo* in HD mice could uncover more robust HD-like phenotypes in these models.

These studies serve to provide new insights into HD modeling and disease mechanisms as well as to identify and validate therapeutic targets for modifying the onset and progression of HD.

## Materials and Methods

### Primary Neuron Culture

Primary neurons were cultured from embryonic day (E)15.5-E17.5 humanized (Hu97/18 and Hu18/18) transgenic mice (Southwell et al., 2013). Brains were removed from embryos and stored in Hibernate-E (Gibco A1247601) overnight during genotyping. The following day, cortices with attached striatal tissue were dissected and pooled by genotype (Hu18/18 or Hu97/18). Cultures were established as in Schmidt et al. (2018). Briefly, tissue was dissociated, trypsinized, and resuspended in Neurobasal complete medium [NBC; 0.02% 10× SM1, 0.01% 100× Pen/Strep, 0.0025% glutamax in Neurobasal Media (Gibco 21103-049)]. The cells were seeded onto poly-D-lysine (PDL) coated plates at a density of 250,000 cells per well in 24-well plates and 1 × 10^6^ cells per well in 6-well plates. For immunocytochemistry, coverslips were treated with hydrochloric acid overnight at room temperature (RT) with gentle shaking and washed in ethanol and phosphate-buffered saline (PBS). Coverslips (Marinefield No. 1.5, 13 mm) were then added to wells and dried before PDL coating. Cells were maintained in a 5% CO_2_/humidified incubator at 37°C. Neurons were fed with 1/10 well volume NBC twice weekly.

### Cell Treatment

For H_2_O_2_, staurosporine, and menadione treatments, a working solution of each oxidative stressor was added at 10× desired final concentration to NBC and given through a neuronal feed of 1/10 well volume. 30% H_2_O_2_ certified ACS stock (Fisher Chemical, H325-100) was diluted into prewarmed NBC, and neurons were treated for 24 h. 5 μM of a 72.4008 mM Menadione stock solution was made in PBS and added to pre-warmed NBC, and neurons were treated for 4 h. A 1 mM stock of staurosporine in DMSO was made, and neurons were treated with 0.5 μM in NBC for 1 h. All conditions were performed in triplicate wells.

### Progerin Treatment

GFP-progerin cDNA was PCR amplified from the pBABE-puro-GFP-progerin plasmid (Addgene, 17663 deposited by Tom Misteli) using primers (forward 5′-GATCATCGATATGGTGAGCAAGGGCGAGGAG-3′, reverse 5′-GATCGCTAGCTTACATGATGCTGCAGTTCTGGG-3′). nGFP was PCR amplified from pAd nGFP (Addgene, 19413, deposited by Douglas Melton) using primers (forward 5′-ATCATCGAT ATGGTGCACGTGGATCCAA-3′, reverse 5′-GATCGCTAGCTTACTTGTACAGCTCGTCCA-3′). Amplified inserts were cloned into the ClaI and NheI sites of the AAV2 backbone vector (pFBAAVCAGmcsBgHpa, G0345) provided by the University of Iowa Viral Vector Core (UIVVC), which generated AAV2/1-GFP-progerin and AAV2/1-nGFP by the baculovirus system. Viral titers of 1.7e13 and 6e12 VG/ml for nGFP and progerin respectively were obtained.

### Cell Death

Cell death was measured in by release of lactate dehydrogenase (LDH) into the media and normalized to total remaining LDH in the lysate using the Pierce LDH Cytotoxicity Kit (Thermo Fisher Scientific, 88953) according to the manufacturer’s specifications.

### Reactive Oxygen Species Detection

ROS was quantified using CM-H2DCFDA (Thermo Fisher Scientific, C6827) according to the manufacturer’s specifications. Briefly, CM-H2DCFDA in DMSO was diluted in NBC and added at a 5 μM concentration to each well. Neurons were protected from light and returned to the incubator for 30 min. In the last 5 min of incubation, Hoescht 3342 (Thermo Fisher Scientific, 62249) was added at 1/1,000 well volume. After washing, ROS fluorescence was visualized and imaged with a Zeiss Axioplan fluorescence microscope. A minimum of three images per well was taken by a researcher blind to genotype and treatment, and integrated optical density (IOD) of fluorescence in images was quantified using ImageJ.

### Primary Neuronal Culture Western Blot

Cells were lysed in the culture dishes in 1/10 well volume SDP lysis buffer [50 mM Tris pH 8.0, 150 mM NaCl, 1% Igepal/NP40, 40 mM β-glycerophosphate (solid), 10 mM NaF and protease inhibitors: 50× Roche Complete, 1× NaVan, 1× PMSF, 1× zVAD]. Dishes were incubated for 30 min with shaking and occasional hand agitation. Lysates were aspirated and cleared by centrifugation at 14,000 rpm for 10 min at 4°C and stored at −80°C until use. A DC assay (BioRad 5000111) was used to quantify lysate protein concentration. 35 μg of total protein was separated by SDS-PAGE using a 4–12% Bis-Tris gel with MOPS running buffer (NuPage). Proteins were then transferred to 0.45 μm nitrocellulose membranes. Membranes were blocked in 5% dry milk powder in PBS for 1 h at RT and incubated overnight at 4°C in primary antibody (anti-lamin B1 1 μg/ml, Abcam ab16048, Cambridge, UK; anti-PCA NCAM 1:500, Millipore MAB5324, Burlington, MA, USA; anti-Dynamin1 1 μg/ml, Abcam ab108458; anti-β-Tubulin 1:5,000, Abcam ab131205; anti-β-Tubulin 1:5,000, Abcam 6046) in 5% BSA in PBS-T. Proteins were detected with IR dye 800CW goat anti-mouse (1:250, Rockland 610-131-007, Gilbertsville, PA, USA) and AlexaFluor 680 goat anti-rabbit (1:250, Molecular Probes A21076, Eugene, OR, USA)-labeled secondary antibodies and the LiCor Odyssey Infrared Imaging System. Densitometry of bands was performed using ImageStudio 4.0 software (Li-Cor Biosciences). Lanes with abnormal loading control were excluded from the analysis.

### YAC128 Striatum Western Blot

Striata from treated YAC128 animals were hand homogenized in SDP buffer. Forty micrograms of total protein was resolved on a 12% gel and transferred onto a 0.45 μm nitrocellulose membrane. Blots were then blocked with 5% skim milk in PBS-T for 1 h and incubated with either rabbit anti-lamin B1 (1:1,000, Abcam, ab16048), rabbit antidynamin 1 (1:1,000, Abcam, ab108458) or mouse anti-CDKN2A (p16INK4a; 1:1,000, Abcam, AB54210) overnight at 4°C. Primary antibodies were detected with IR dye 800CW goat antimouse (1:5,000, Rockland, 610-131-007) and AlexaFluor 680 goat anti-rabbit (1:5,000, Molecular Probes, A21076)-labeled secondary antibodies. Blots were subsequently probed with mouse anti-β-Tubulin (1:5,000 Abcam, ab6046) as a loading control and detected with IR dye 800 CW goat anti-mouse. Proteins were visualized and quantified as above.

### iPSC Differentiation and Treatment

iPSC generation was previously performed at Cedar’s Sinai Medical Center using non-integrating methods from human fibroblast lines obtained from three HD patients with CAG repeat sizes of 43 (CS13iHD43n2), 71 (CS81iHD71n), or 109 (CS09iHD109n), and from three non-HD control subjects with CAG repeat sizes of 18 (CS25iCTR18n), 21 (CS00iCTR21n), or 33 (CS83iCTR33n; The HD iPSC Consortium, 2012; Mattis et al., 2015; Mehta et al., 2018). These iPSCs were fully reprogrammed as previously demonstrated by testing for pluripotency markers and gene expression analysis (Mattis et al., 2015), and removal of reprogramming plasmids was confirmed by genomic polymerase chain reaction (PCR) and Southern blotting (Mattis et al., 2015). Neural stem cells (NSCs) were created using previously published protocols (Telezhkin et al., 2016; Garcia et al., 2019). NSCs were cryopreserved at 16 days of differentiation in DMSO-supplemented cell freezing media (Sigma, C6164) as described (Ebert et al., 2013). Coverslips were prepared by washing in HCl overnight, followed by washing 3× in ethanol and 3× in 1× PBS. Once dry, coverslips were transferred to the bottom of 24-well plates and coated with Corning^®^ Matrigel^®^ Matrix. NSCs were thawed onto coverslips and directed toward a striatal fate by plating with neuronal induction media supplemented with GABA and pro-synaptogenic small molecules (CHIR99021 and forskolin), as previously published (Kemp et al., 2016; Telezhkin et al., 2016; Garcia et al., 2019). NSCs were infected with AAV2/1-nGFP or AAV2/1-progerin-GFP at 1e10 VG/ml the following day. Triplicate wells were used for each condition. Neurons were directed toward a striatal fate for an additional 9 days with an expression of progerin or nGFP, upon which neurons were fixed, stained, and transferred to slides using ProLong^TM^ Gold Antifade Mountant with DAPI (Thermo Fisher Scientific). Two sets of neuron staining combinations were performed. The first set was stained for a marker of neuronal dendrites (MAP2 1:250, Sigma–Aldrich m1408, St. Louis, MO, USA), a striatal neuron marker (DARPP-32 1:400, Cell Signaling 19A3, Beverly, MA, USA), and viral transduction (GFP 1:2,000, Abcam ab13970, Cambridge, UK). The second set was stained for a marker of apoptosis (cleaved caspase-3), DNA damage (γ-H_2_AX 1:1,000, Upstate Biotechnology), and viral transduction (GFP 1:2,000, Abcam ab13970). Neurons were imaged using a Zeiss Axio Observer.Z1 confocal microscope at 40× and 63× objective magnification and were background-corrected using ImageJ with a rolling ball radius of 35 pixels. Control (CAG18, CAG21, and CAG33) and HD (CAG43, CAG71, CAG109) were pooled for analyses.

### Dendritic Analysis

The dendritic analysis was performed as described (Schmidt et al., 2018). Briefly, fluorescence images of cells stained with a marker of neuronal dendrites (MAP2 1:250, Sigma–Aldrich m1408) were acquired using a Leica TCS SP8 confocal laser scanning microscope at 63× objective magnification. Samples from different groups were interleaved and the researcher was blinded to experimental conditions during imaging and analysis. Image stacks of the *z*-step size of 60 μm were converted to 2D in Image J using the maximum intensity *z-projection* function. Images were then background subtracted with a rolling ball radius of 35 pixels and de-speckled. Images were imported into NeuronStudio (Version 0.9.92) for semi-automated measurement of dendritic length and Sholl analysis.

### Immunocytochemistry

Neurons grown on coverslips were fixed with 4% PFA for 20 min at room temp, diluted in 1xPBS washed in 1× PBS, and blocked in 3% BSA, 5% normal goat serum and 0.15% triton in PBS for 20 min at RT. Fixed cells were then incubated overnight at 4°C in primary antibody (GFP 1:2,000, Abcam ab13970, γH2A.X 1:500, Sigma–Aldrich 05-636; Map-2 1:250, Sigma–Aldrich M4403; Map-2 1:250, Invitrogen PA517646; EM48 1:100, Millipore MAB5374; LC3B 1:2,000, Novus Biologicals NB600-1384 Centennial, CO, USA; DNA/RNA damage [15A3] 1:500 Abcam ab62623; DARPP-32 1:500, Abcam ab40801; Cleaved caspase-3 1:400, Cell Signal 9664 Danvers, MA; TUJ-1 1:1,000, Sigma–Aldrich T8660; Nestin 1:500 Millipore MAB5326), washed 3× with PBS, and subsequently incubated in secondary antibody (goat anti-chicken 488 1:200, Invitrogen A32931; goat anti-rabbit 647 1:200, Invitrogen A21244; goat anti-rabbit 568 1:200, Invitrogen A11011; goat anti-mouse 647, Invitrogen A21235 1:200; goat anti-mouse 568 1:200, Invitrogen A11004) for 1 h at RT protected from light. After washing 3× with PBS, coverslips were mounted on 75 mm slides (Fisherbrand, 12-550-15) with ProLong Gold Antifade mounting reagent with DAPI. Stained cells were imaged with a Zeiss Axioplan fluorescence microscope with 20×, 40×, and 63× magnification objectives. At least three images were taken per well and integrated density/fluorescence was quantified using ImageJ.

### Surgical AAV Delivery

AAVs were delivered by stereotaxic intrastriatal convection-enhanced delivery as in Southwell et al. (2009). Briefly, a burr hole was made at 0.8 mm anterior and 2 mm lateral to Bregma. A Hamilton syringe with 30-gauge needle preloaded with 4 μl sterile saline and 1 μl virus was slowly lowered to 3.5 mm below the dura. A microinjector was then used to inject the virus followed by the bolus of sterile saline at a rate of 0.5 μl/min. The needle was left in place for 5 min and slowly withdrawn.

### Tissue Collection and Processing

For mice allotted for terminal molecular and biochemical analysis, brains were removed and placed on ice for 1 min to increase tissue rigidity. Brains were then microdissected by region. Striata and cortices were preserved in RNAlater (Ambion AM7021) overnight at 4°C and then stored at −80°C until use. Mice allotted for terminal histological analysis were perfused transcardially with PBS and 4% PFA. Brains were removed and post-fixed in 4% PFA in PBS for 24 h at 4°C. The following day brains were cryoprotected in 30% sucrose with 0.01% sodium azide. Once equilibrated, brains were divided into forebrain and cerebellum, and forebrains were frozen on dry ice, mounted in the Tissue-TEK O.C.T. embedding compound (Sakura), and cut *via* cryostat (Leica CM3050S) into a series of 25 μm coronal sections free-floating in PBS with 0.01% sodium azide.

### Brain Histology

For evaluation of viral distribution, a series of sections spaced 200 μm apart, and spanning the striatum were stained with primary rabbit anti-GFP (1:1,000, Life Technologies) and secondary goat anti-rabbit (1:500, Alexa-fluor 488). Sections were mounted using ProLong Gold Antifade mounting reagent (Thermo Fisher P36930) with DAPI (Life Technologies) and imaged with a 2.5× objective (ZEISS Microscopy) using a Zeiss Axio Vert.A1 microscope (ZEISS Microscopy), Zeiss AxioCam ICm1 camera (Carl Zeiss), and Zeiss Zen 2.3 Lite software (Carl Zeiss). Four images per section were merged using the PhotoMerge tool in Adobe Photoshop CC 2018 (Adobe).

To evaluate viral tropism and progerin-induced changes, free-floating sections were blocked for 0.5 h at RT in 5% normal goat or normal donkey serum with 0.1% Triton X-100 in PBS. Primary antibodies were incubated overnight at RT and secondary antibodies were incubated for 2 h at RT. A series of sections spaced 200 μm apart and spanning the striatum were co-stained with rabbit anti-GFP (1:1,000, Life Technologies) primary and donkey anti-rabbit (1:500, Alexafluor 488) secondary antibody as well as either mouse/rat anti-DARPP-32 (1:1,000, R&D Systems MAB4230) primary and goat anti-rat (1:500, Alexa-fluor 568) secondary antibody, mouse anti-NeuN (1:1,000, Millipore MAB377) primary and goat anti-mouse (1:500, Alexa-fluor 568) secondary antibody or mouse anti-GFAP CY3 conjugate (1:500, Sigma C9205). Additional sections were stained with either P16INK4a (1:500, ab54210) or 8-Oxo-dG (1:500, R&D systems 4354-MC-050) primaries and goat anti-mouse (1:500, Alexa-fluor 568) secondary antibody. The sections were mounted using ProLong Gold antifade mounting reagent with DAPI (Life Technologies). Sections were imaged with a 63× objective on a Leica SP5 laser scanning confocal microscope. *z*-stacks were performed with a step size of 0.5 μm and separate color channels were imaged sequentially in a 1,024 × 1,024 pixel array with a scan speed of 400 Hz and a pinhole diameter of 90 μm.

### Statistical Analysis

Statistical analyses were performed in Graphpad Prism (v. 8.1.1). Two way analysis of variance (ANOVA) with Bonferroni *post hoc* correction was performed to determine differences between treatments and genotypes or unpaired *t*-tests for comparison of only two groups. For image quantification, images with fewer than three neurons (determined by DAPI quantification) were excluded from analyses.

## Results

### Exogenous Oxidative Stress Does Not Induce ROS in Immature HD Neurons

**Figure 1:**
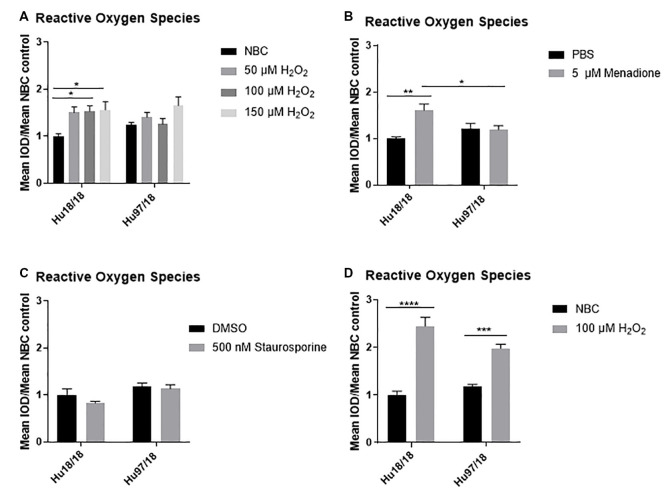
Oxidative stressors increase reactive oxygen species (ROS) in immature control and mature Huntington’s Disease (HD) primary neurons. Primary Hu97/18 and Hu18/18 control neurons were treated with stressors for 24 h and ROS was measured by live-imaging of CM-H2DCFDA fluorescence. Immature neurons were treated with **(A)** neurobasal complete (NBC) or H_2_O_2_ at the indicated doses. **(B)** PBS or 5 μM menadione. **(C)** DMSO or 500 nM staurosporine. **(D)** Mature neurons were treated with 100 μM H_2_O_2_. All data were normalized to mean Hu18/18 vehicle control. *Difference between indicated bars, *p < 0.05, **p < 0.01, ***p < 0.001, ****p < 0.0001. Error bars ± SEM.

To induce oxidative stress in primary forebrain neurons from Hu97/18 HD and Hu18/18 control mice, we treated neurons with either neurobasal complete (NBC) media containing hydrogen peroxide (H_2_O_2_), which is taken up by neurons and increases ROS, or NBC vehicle. To ensure H_2_O_2_ was inducing oxidative stress, we first measured ROS, 24 h post-treatment. We found that H_2_O_2_ induced ROS in control neurons (Figure 1A; two way ANOVA treatment *p* = 0.0021, genotype 0.8977, Bonferroni post-test different from NBC Hu18/18: 50 μM, *p* = 0.0773; 100 μM, *p* = 0.0133; 150 μM, *p* = 0.0227), but failed to induce ROS in HD neurons (Different from NBC Hu97/18 18 50 μM, *p* = 0.6155; 100 μM, *p* = 0.9986; 150 μM, *p* = 0.0390). To determine whether this unexpected effect was H_2_O_2_ specific, we treated neurons with menadione or staurosporine, two compounds that also increase ROS in neurons. We found a similar effect in menadione-treated neurons, with the induction of ROS in control, but not HD, neurons (Figure 1B, treatment *p* = 0.0196, genotype *p* = 0.4051, post-test vs. PBS same genotype Hu18/18: *p* = 0.0050; Hu97/18: *p* > 0.9999). However, menadione treatment also caused nuclear abnormalities in neurons in the absence of elevated cell death (Supplementary Figures S1A,B). Treatment with staurosporine, a non-selective protein kinase inhibitor, failed to induce ROS in control or HD neurons (Figure 1C, treatment *p* = 0.2258, genotype *p* = 0.0090, post-test vs. DMSO same genotype Hu18/18: *p* = 0.3554; Hu97/18: *p* > 0.9999). It is interesting to note that although staurosporine did not induce ROS in neurons, it did cause cell death selectively in HD neurons at 1 μM (Supplementary Figure S1C, post-test different from NBC Hu97/18, 1,000 nM *p* < 0.0001, all other comparisons ns), suggesting that high levels of kinase inhibition are not well-tolerated by HD neurons. Due to the nuclear abnormalities observed with menadione treatment and the lack of ROS-induction with staurosporine treatment, we have used H_2_O_2_ treatment to induce oxidative stress in all subsequent experiments.

These initial experiments were conducted at the day *in vitro* (DIV)13, a time-point frequently used in primary neuron culture. However, primary neurons do not begin to reach full maturity and become maximally electrically active until DIV21. Because of this, we repeated our initial experiments in neurons at DIV21. Contrary to what we saw in immature HD neurons, mature HD neurons exhibited the expected increase in ROS upon H_2_O_2_ treatment (Figure 1D, two way ANOVA treatment *p* < 0.0001, genotype *p* = 0.2505, post-test vs. NBC same genotype Hu18/18: *p* < 0.0001; Hu97/18: *p* = 0.0003). This demonstrates that mature neurons may more accurately model cellular dysfunction in adult-onset diseases.

### Mature HD Neurons Have Elevated Basal Oxidative Damage That Is Not Exacerbated by H_2_O_2_ Treatment

**Figure 2:**
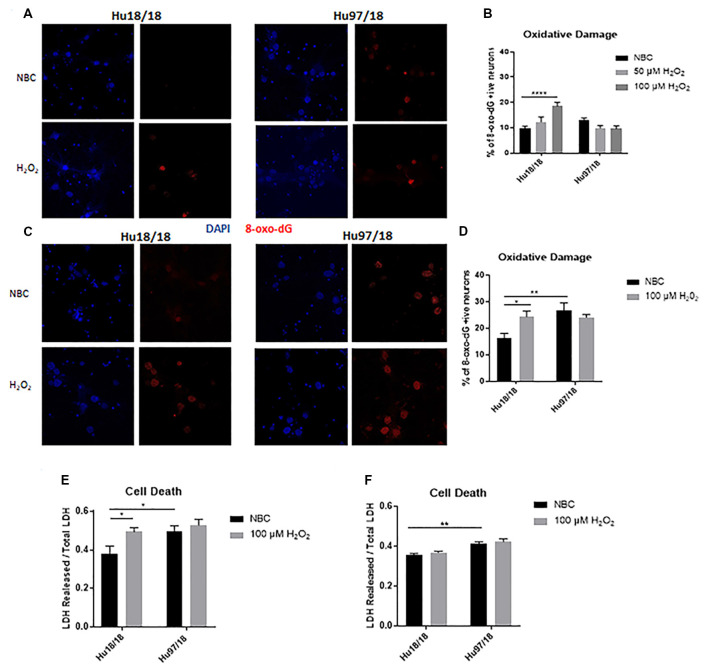
Oxidative stress does not induce oxidative damage or cell death in HD primary neurons. Primary Hu18/18 and Hu97/18 neurons were treated with NBC vehicle or 100 μM H_2_O_2_ for 24 h. **(A–D)** DNA damage was assessed by 8-Oxo-dG immunocytochemistry. **(A,B)** Representative images **(A)** and quantification **(B)** of % 8-Oxo-dG positive immature neurons. **(C,D)** Representative images **(C)** and quantification **(D)** of % 8-Oxo-dG positive mature neurons. **(E,F)** Cell death was assessed by quantifying LDH released/total in **(E)** immature and **(F)** mature neurons. *Difference between indicated bars, *p < 0.05, **p < 0.01, ****p < 0.0001. Error bars ± SEM.

To interrogate whether HD neurons are hypersensitive to oxidative stress, we treated control and HD neurons with H_2_O_2_ or NBC vehicle control for 24 h and measured residual oxidative damage by quantifying 8-Oxo-2′-deoxyguanosine (8-Oxo-dG), a marker of DNA oxidation. We found an increase in oxidative damage in immature control, but not HD neurons when treated with H_2_O_2_ (Figures 2A,B, two way ANOVA treatment *p* = 0.0420, genotype *p* = 0.0101, post-test vs. NBC same genotype Hu18/18: 50 μM, *p* = 0.3024, 100 μM, *p* < 0.0001; Hu97/18: 50 μM, *p* = 0.1877, 100 μM, *p* = 0.1467). Because oxidative damage was significantly elevated in Hu18/18 neurons at 100 μM, this dose was chosen for subsequent experiments. Interestingly, we observed similar effects in mature neurons; oxidative damage was induced in mature control, but not HD neurons (Figures 2C,D, two way ANOVA treatment *p* = 0.0140, genotype *p* = 0.1973, post-test vs. NBC same genotype Hu18/18 *p* = 0.0140; Hu97/18 *p* = 0.5905). However, we did observe elevated basal levels of oxidative DNA damage in mature, but not immature HD neurons (Figures 2B,D, post-test vs. NBC Hu18/18 same DIV, immature *p* = 0.3186, mature *p* = 0.0010), resulting in damage similar to that observed in H_2_O_2_ treated control neurons.

To determine whether the absence of observed H_2_O_2_-induced DNA damage in HD neurons resulted solely from increased cell death, we measured lactate dehydrogenase (LDH) release in basal and H_2_O_2_ treated conditions. We found H_2_O_2_ treatment caused significant cell death in immature control, but not HD neurons (Figure 2E, treatment *p* = 0.0220, genotype *p* = 0.0213, post-test vs. NBC same genotype Hu18/18: *p* = 0.0270; Hu97/18: *p* = 0.8742). However, basal levels of cell death were increased in immature HD neurons (Figure 2E, post-test vs. NBC Hu97/18 *p* = 0.0345). In mature neurons, H_2_O_2_ did not induce cell death in control or HD neurons. However, HD neurons displayed increased levels of basal cell death (Figure 2F, treatment *p* = 0.2289, genotype *p* < 0.0001, post-test Hu97/18 NBC vs. Hu18/18 NBC *p* = 0.0011).Together, these data demonstrate that mature HD primary neurons have some mild phenotypes which are not exacerbated by exogenous oxidative stress.

### H_2_O_2_ Treatment Does Not Induce Canonical Stress Response Pathways in HD Neurons

**Figure 3:**
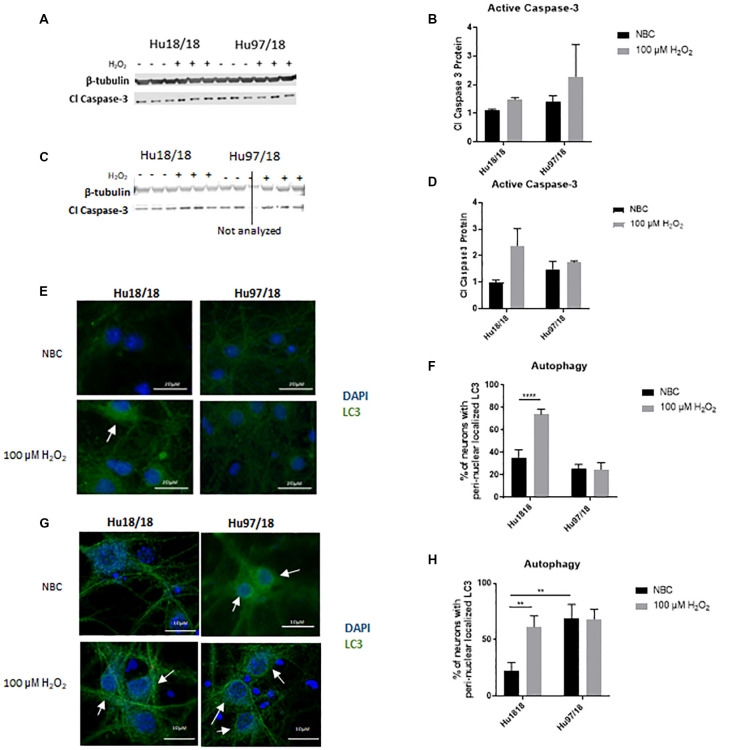
H_2_O_2_ induces autophagic and possibly caspase-3 apoptotic pathways in control but not HD neurons. Primary Hu18/18 and Hu97/18 neurons were treated with NBC vehicle or 100 μM H_2_O_2_ for 24 h. **(A–D)** Apoptosis was assessed by quantifying active caspase-3 protein by WB. **(A,B)** Representative images **(A)** and quantification **(B)** in immature neurons. **(C,D)** Representative images **(C)** and quantification **(D)** in mature neurons. **(E–G)** Autophagy was assessed by LC3 immunocytochemistry. **(E,F)** Representative images **(E)** and quantification **(F)** of % of immature neurons with perinuclear localized LC3. **(G,H)** Representative images **(G)** and quantification **(H)** of % of mature neurons with perinuclear localized LC3. *Difference between indicated bars, **p < 0.01, ****p < 0.0001. Error bars ± SEM.

Caspase-3 is a protease that, when activated, initiates the cellular apoptosis cascade, which can be the ultimate result of oxidative stress (Carvour et al., 2008). To determine if caspase-3 signaling is altered in HD neurons or in response to H_2_O_2_ treatment, we measured active caspase-3 protein. We found that in immature neurons, caspase-3 was not induced in control or HD neurons after H_2_O_2_ treatment. However, in mature control neurons, we saw a strong trend toward increased activation of caspase-3 post- H_2_O_2_ treatment, suggesting a potential induction of caspase-3 activation. HD neurons did not exhibit this trend (Figures 3A–D, Immature neurons: treatment *p* = 0.2988, genotype *p* = 0.3600, post-test vs. NBC same genotype Hu18/18 *p* > 0.9999, Hu97/18 *p* = 0.6149; mature neurons: treatment *p* = 0.2988, genotype *p* = 0.36, post-test vs. NBC same genotype Hu18/18 *p* = 0.0603, Hu97/18 *p* > 0.9999), suggesting that HD neurons do not activate caspase-3 dependent apoptosis in response to oxidative stress.

Upstream of apoptosis is the autophagy pathway, which recycles proteins and organelles to decrease damage in cells. To investigate the autophagic response in HD primary neurons, we treated control and HD neurons with H_2_O_2_ or NBC vehicle control and assessed perinuclear localization of LC3-positive autophagic vesicles, which organize in a perinuclear fashion in response to stress (Filomeni et al., 2015). We found increased perinuclear LC3 localization in response to H_2_O_2_ treatment in immature control, but not HD neurons (Figures 3E,F, treatment *p* = 0.0016, genotype *p* < 0.0001, post-test vs. NBC same genotype Hu18/18 *p* < 0.0001, Hu97/18 *p* > 0.9999). Similarly, in mature neurons, we observed induction of perinuclear LC3 localization in control neurons, and no effect in HD neurons (Figures 3G,H, treatment *p* = 0.0460, genotype *p* = 0.0057, post-test vs. NBC same genotype Hu18/18 *p* = 0.0056, Hu97/18 *p* > 0.9999). However, we also saw increased basal perinuclear LC3 in mature HD neurons (Hu97/18 NBC vs. Hu18/18 NBC *p* = 0.0018), demonstrating dysregulated autophagy only in mature HD neurons.

### Progerin Treatment Induces Aging-Related Phenotypes and Enhances Stress-Related Phenotypes in HD Primary Neurons

Because primary neurons, even when matured in culture, are still developmental, we asked whether ‘aging’ neurons *in vitro* would uncover more robust cellular phenotypes. To induce age-associated changes in mature neurons, we treated neurons with progerin, using an adeno-associated virus (AAV2/1-GFP-progerin) or nuclear GFP control (AAV2/1-nGFP). To account for the 4 days required to achieve maximal transgene expression and for consistency with a previous study using 5 days of progerin expression to age PD iPSC-derived neurons (Miller et al., 2013), neurons were assessed 9 days post-infection. In preliminary dosing experiments, we used iPSC colonies grown feeder-free treated with AAV2/1-nGFP. We observed efficient transduction and high transgene expression after this interval only in cells spontaneously differentiating in iPSC colonies, but not in undifferentiated iPSCs (Supplementary Figure S2A). In a dosing study in neurons directed toward striatal fate from neural progenitors using GABA and prosynaptogenic factors, we found efficient transduction at e9–e10 viral genomes/ml (VG/ml; Supplementary Figure S2B), and therefore this dose range was used for all subsequent experiments.

**Figure 4:**
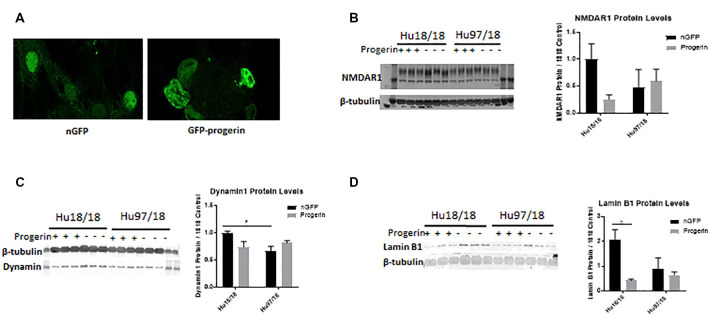
Progerin treatment induces aging in primary neurons. Mature primary Hu18/18 and Hu97/18 neurons were treated with nGFP or progerin. **(A)** Progerin induces nuclear blebbing, which occurs with age. **(B–D)** WB analysis of proteins known to decrease over natural aging; **(B)** NMDAR1, **(C)** Dynamin1, and **(D)** lamin B1. *Difference between indicated bars, *p < 0.05. Error bars ± SEM.

**Figure 5:**
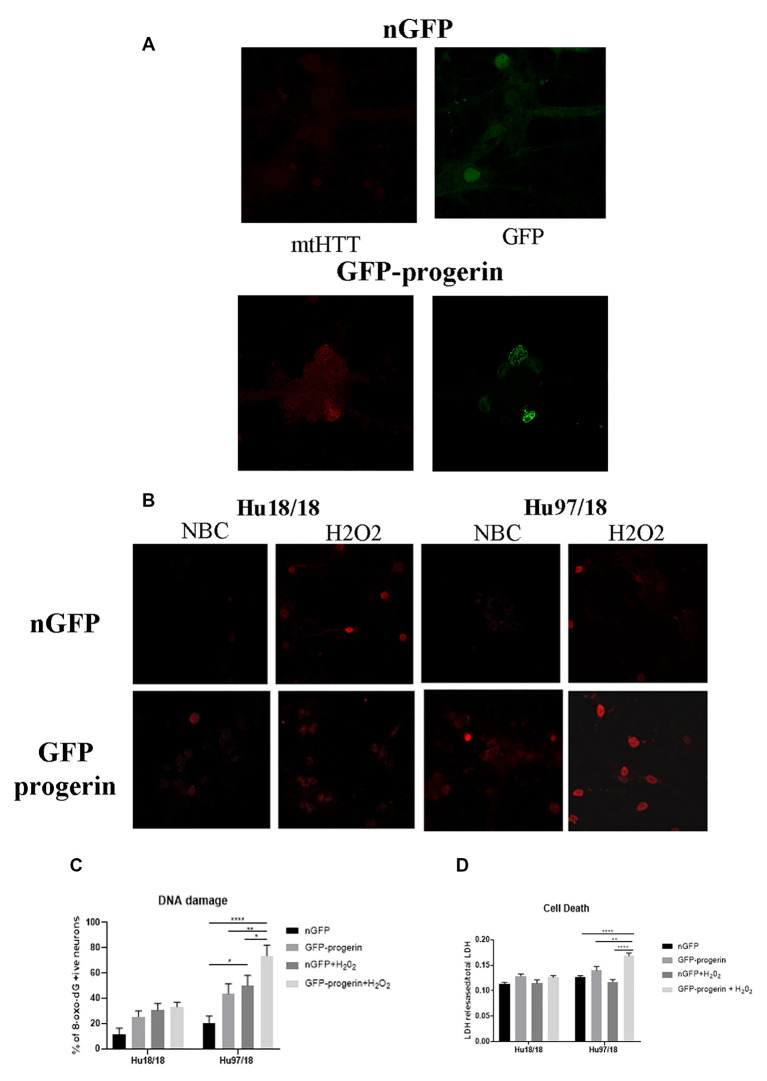
Progerin treatment causes aggregate seeding/oligomerization of mutant HTT (mtHTT) and increased susceptibility to oxidative stress-induced DNA damage and cell death in HD neurons. **(A)** Mature Hu18/18 and Hu97/18 neurons were treated with nGFP or progerin to induce aging, and mtHTT was assessed by EM48 immunocytochemistry. **(B,C)** Mature Hu18/18 and Hu97/18 neurons were aged with progerin or given nGFP control followed by H_2_O_2_ or vehicle control treatment. Oxidative DNA damage was assessed by Oxo-8-dG immunocytochemistry. Representative images **(B)** and quantitation of % of neurons exhibiting DNA damage **(C)**. **(D)** Mature primary Hu18/18 and Hu97/18 neurons were treated with nGFP or progerin followed by H_2_O_2_ or vehicle control. Cell death was assessed by quantifying released/total lactate dehydrogenase (LDH). *Difference between indicated bars, *p < 0.05, **p < 0.01, ****p < 0.0001. Error bars ± SEM.

We first investigated whether progerin could induce markers of age in primary forebrain neurons. We found that progerin treatment caused nuclear blebbing, which can be associated with apoptosis, but is also a sign of cellular senescence, in neurons irrespective of genotype; this was not observed in nGFP-treated control or HD neurons (Figure 4A). However, this is not surprising considering lamin A is a nuclear lamin protein. We also quantified proteins known to change with age. We found that progerin treatment caused a trend toward decreased NMDAR1 and dynamin 1 levels in control, but not HD neurons (Figures 4B,C, NMDAR1 treatment *p* = 0.2436, genotype *p* = 0.7193, post-test vs. nGFP same genotype Hu18/18 *p* = 0.1341, Hu97/18 *p* > 0.9999; dynamin 1 treatment *p* = 0.4405, genotype *p* = 0.1068, post-test vs. nGFP same genotype Hu18/18 *p* = 0.0611, Hu97/18 *p* = 0.3572). We also saw a dramatic decrease in lamin B1, which is the primary nuclear lamina protein in neurons, in progerin-treated control neurons (Figure 4D, treatment *p* = 0.0183, genotype *p* = 0.1711, post-test vs. nGFP same genotype Hu18/18 *p* = 0.0129, Hu97/18 *p* > 0.9999). Interestingly, we also saw those HD neurons basally had a trend toward decreased NMDAR1 (Figure 4B, Hu97/18 nGFP vs. Hu18/18 nGFP *p* = 0.3485), a significant decrease in dynamin 1 (Figure 4C, Hu97/18 nGFP vs. Hu18/18 nGFP *p* = 0.03572), and a strong trend toward decreased lamin B1 (Figure 4D, Hu97/18 nGFP vs. Hu18/18 nGFP *p* = 0.0605). We also noted that in mature neurons, treatment with progerin caused a cellular rearrangement of HD neurons, which is a stress response (Supplementary Figure S3). Together, this demonstrates that progerin treatment alters nuclear architecture and proteins associated with neuronal aging. Furthermore, mature HD neurons display some alterations in proteins associated with neuronal aging at a basal level, further supporting accelerated biological age in HD neurons. Oligomerization and aggregation of mtHTT is a characteristic of HD brains and neurons, but something that is not observed in embryonic HD primary neurons. We assessed mtHTT aggregation by EM48 immunoreactivity, an anti-HTT antibody that recognizes only aggregated or oligomeric mtHTT. Mature HD primary neurons entirely lack EM48 immunoreactivity, suggesting that mtHTT is primarily in a soluble, monomeric state within these cells. However, after inducing age-associated changes by progerin treatment, we observed EM48 immunoreactivity (Figure 5A), suggesting that mtHTT has undergone some form of aggregate seeding and/or oligomerization. Thus, suggesting that biological age can modulate the folding and aggregation state of mtHTT protein irrespective of the passage of time.

We next investigated how progerin treatment affects sensitivity to oxidative stress by treating neurons with nGFP (nGFP + vehicle), progerin (GFP-progerin + vehicle), H_2_O_2_ (nGFP + H_2_O_2_), or the combination (progerin + H_2_O_2_). We found a strong trend toward a selective increase in oxidative DNA damage in HD, but not control, neurons when treated with progerin (Figures 5B,C, Supplementary Figures S4, S5, treatment *p* < 0.0001, genotype *p* < 0.0001, post-test vs. nGFP same genotype Hu18/18 progerin *p* = 0.8699; Hu97/18 progerin *p* = 0.1088. Similar results were seen for cells treated with H_2_O_2_ (post-test vs. nGFP same genotype Hu18/18 H_2_O_2_ *p* = 0.1919; Hu97/18 H_2_O_2_ *p* = 0.0131). Interestingly, we found the combination of progerin and oxidative stress to synergistically increase oxidative damage selectively in HD neurons beyond what was seen with either insult alone (post-test vs. progerin + H_2_O_2_ same genotype Hu18/18 nGFP *p* = 0.0733, progerin *p* > 0.9999, H_2_O_2_ *p* > 0.9999; Hu97/18 nGFP *p* < 0.0001; progerin *p* = 0.0069, H_2_O_2_ *p* = 0.0500). Additionally, we found that the combination of progerin treatment and H_2_O_2_ mediated oxidative stress did not affect cell death in control neurons. Conversely, in HD neurons, this combination did induce cell death where either insult alone did not (Figure 5D, treatment *p* < 0.0001, genotype *p* < 0.0001, post-test vs. nGFP same genotype Hu18/18 progerin *p* = 0.1679, H_2_O_2_ *p* > 0.9999, progerin + H_2_O_2_ *p* = 0.3909; Hu97/18 progerin *p* = 0.3781, H_2_O_2_ *p* > 0.9999, progerin + H_2_O_2_ *p* < 0.0001). Together these results demonstrate that progerin treatment, which induces age-like changes, enhances HD-related phenotypes, and that there is a potential synergistic effect of aging and cellular stress on dysfunction and death of HD primary neurons.

### Inducing Age-Related Changes With Progerin Enhances HD Phenotypes in iPSC-Derived Human Neurons

**Figure 6:**
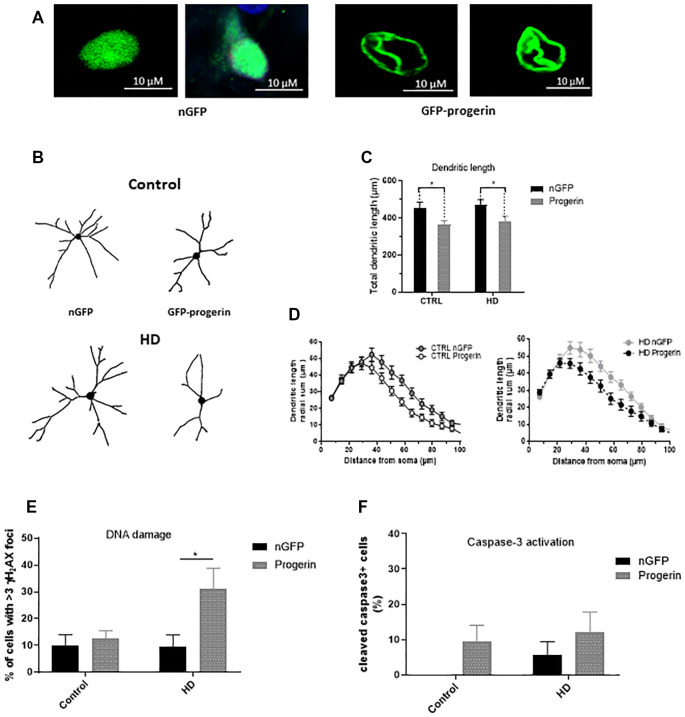
Progerin treatment induces age-related changes and enhances HD-like phenotypes in HD induced pluripotent stem cell (iPSC) derived neurons. HD patient and healthy control iPSCs were differentiated toward striatal-like neurons and treated with nGFP or progerin and evaluated by immunocytochemistry. **(A)** GFP staining demonstrates nuclear blebbing in progerin, but not nGFP treated neurons. **(B–D)** Dendritic morphology was assessed in transduced cells by **(C)** total dendrite length and **(D)** radial dendritic complexity. **(E)** Apoptosis was assessed by % of transduced neurons that are caspase-3 positive. **(F)** DNA damage was assessed by % of transduced neurons with >3 γH_2_AX foci, revealing a selective increase in HD neurons. *Difference between indicated bars. *p < 0.05. Error bars ± SEM.

To investigate if inducing age-related changes with progerin could enhance HD-like phenotypes in patient iPSC-derived neurons similar to what we observed in primary neurons, we infected HD (CAG43, CAG71, CAG109) and control (CAG18, CAG21, CAG33) neural progenitors with AAV2/1-GFP-progerin or AAV2/1-nGFP during BDNF-dependent differentiation toward a striatal-like fate. Similar to primary neurons, we found that progerin treatment caused nuclear blebbing in neurons irrespective of the genotype that was not observed in response to treatment with nGFP (Figure 6A). We also found that progerin treatment decreased dendritic length and radial dendritic complexity of neurons (Figures 6B–D, treatment *p* = 0.0006, genotype *p* = 0.5275, post-test vs. nGFP same genotype Hu18/18 *p* = 0.0294, Hu97/18 *p* = 0.0280). This could be indicative of reduced systems-level connectivity. Similar to primary neurons, we observed a trend toward caspase 3 activation in response to progerin treatment in neurons (Figure 6E, treatment *p* = 0.1060, genotype *p* = 0.4132, post-test vs. nGFP same genotype Hu18/18 *p* = 0.4182, Hu97/18 *p* = 0.6036), indicating that our aging paradigm itself does not induce the apoptotic cascade.

We also assessed DNA damage in neurons by immunocytochemistry of γH_2_AX foci. Similar to what we observed in primary neurons, we measured selectively increased DNA damage in progerin-treated HD neurons (Figure 6F, treatment *p* = 0.0514, genotype *p* = 0.1383, post-test vs. nGFP same genotype Hu18/18 *p* > 0.9999, Hu97/18 *p* = 0.0190) that was not seen with nGFP treatment, thus further supporting exacerbation of HD-like changes in aged neurons.

### Progerin Treatment Induces Age-Related Changes and Oxidative Damage in the Brain of HD Mice

Finally, we evaluated if the expression of progerin in the brains of HD mice could induce aging-related phenotypes and exacerbate HD-related phenotypes as was observed in primary and iPSC-derived neurons. YAC128 HD model mice received bilateral intrastriatal injections with 1e10 VG/hemisphere of either AAV2/1-GFP-progerin or AAV2/1-GFP at 2 months of age and were collected 8 weeks post-injection for immunohistochemical or Western blot analysis.

**Figure 7:**
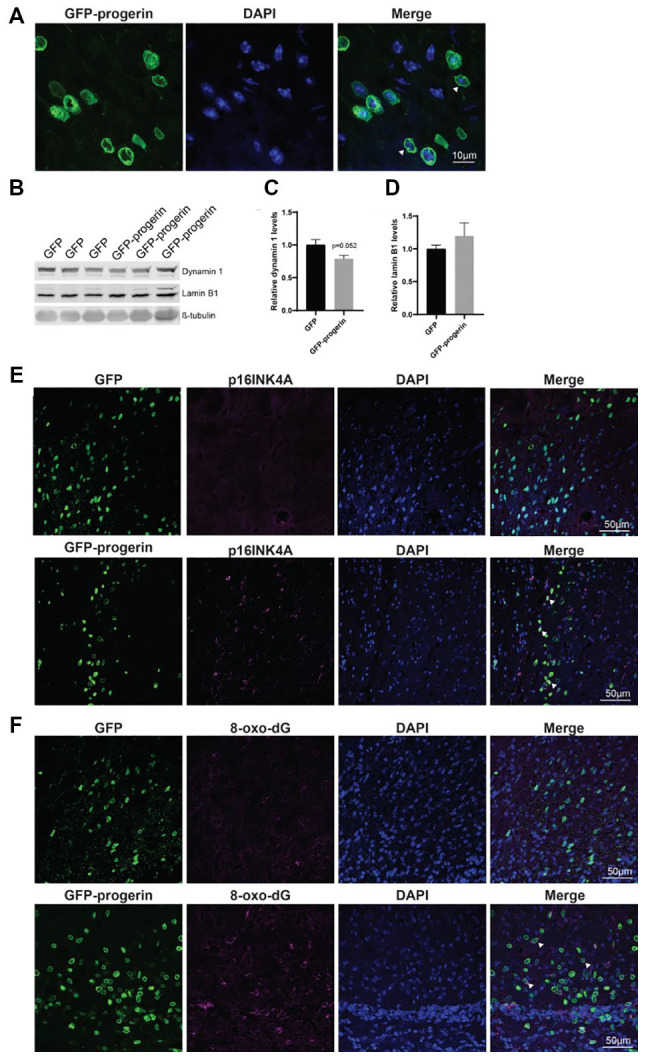
Progerin treatment induces aging and DNA damage in the HD mouse brain.** (A)** Representative confocal image of the YAC128 brain treated with AAV2/1-GFP-progerin shows nuclear blebbing. Scale bar = 10 μm. Representative Western blots **(B)** and quantifications of aging markers **(C)** dynamin 1 and **(D)** lamin B1. Error bars ± SEM. **(E,F)** Confocal images of **(E)** p16^INK4A^ or **(F)** 8-Oxo-dG staining in the YAC128 mouse brain treated with either GFP or GFP-progerin.

Broad distribution throughout the striatum and deeper layers of the cortex was observed with both AAV2/1-GFP (Supplementary Figure S6A) and AAV2/1-GFP-progerin (Supplementary Figure S6B). Consistent with the reported tropism of this AAV serotype, we observed efficient transduction of MSNs (Supplementary Figure S7A), neurons (Supplementary Figure S7B), as well as astrocytes and neural progenitors (Supplementary Figure S7C) in the striatum. Similar to our culture models, nuclear blebbing was observed in cells expressing GFP-progerin (Figure 7A).

To evaluate the effect of progerin on markers of aging in YAC128 mice, striatal tissues from GFP and GFP-progerin treated mice were assessed for dynamin 1 and lamin B1 by Western blot (Figure 7B). We observed a strong trend towards a decrease in dynamin 1 levels in YAC128 mice in response to progerin-induced aging but this did not reach significance (Figures 7B,C, unpaired *t*-test *p* = 0.052). We did not observe any effect of progerin treatment on lamin B1 levels (Figures 7B,D, unpaired *t*-test *p* = 0.3630). These data suggest that progerin can induce selected markers of aging in the brain of YAC128 mice. It is important to note that lysates represent an average of all the cells in that tissue and that the effects of progerin treatment on levels of aging makers in these striatal lysates may be blunted as a result of cells that were not transduced. To overcome this, we performed qualitative immunohistochemistry using the aging marker p16^INK4a^ (Baker et al., 2008), following progerin treatment. We observed negligible p16^INK4a^ staining in the striatum of YAC128 mice treated with AAV2/1-GFP. Conversely, we observed strong staining for p16^INK4a^ in the striatum of AAV2/1-GFP-progerin treated YAC128 mice (Figure 7E).

We also qualitatively assessed the effect of progerin on oxidative DNA damage in YAC128 brains by 8-Oxo-dG immunoreactivity, which labels oxidized deoxyguanosine nucleotides. Similar to p16^INK4a^, 8-Oxo-dG was only minimally observed in the striatum of YAC128 mice treated with AAV2/1-GFP but was robustly observed in the striatum of AAV2/1-GFP-progerin treated YAC128 mice (Figure 7F). There was also a high degree of overlap between 8-Oxo-dG and progerin transduced cells, indicating that progerin treatment induces oxidative DNA damage in the brain of HD mice.

## Discussion

Here, we demonstrate differences in the oxidative stress response between immature and mature primary forebrain neurons from HD mice. Increased DNA damage has been reported in HD (Browne et al., 1999; Polidori et al., 1999; Bogdanov et al., 2001; Chen et al., 2007; Sorolla et al., 2008; Brocardo et al., 2016). The antioxidant and DNA damage response has also been reported to be diminished in HD neurons (Beal et al., 1992; Chen et al., 2007; Peña-Sáanchez et al., 2015). Surprisingly, we observed decreased susceptibility to oxidative stress in immature HD neurons compared to control or mature HD neurons. This is an important distinction because previous studies investigating the role of stress in HD in primary neurons have been performed in neurons at DIV13 (Zeron et al., 2002; Graham et al., 2009; Tsvetkov et al., 2010). However, primary neurons do not begin to reach maturity and become maximally electrically active until DIV21 (Biffi et al., 2013), which could potentially skew conclusions drawn from these studies. We found that while Hu97/18 immature neurons had slightly elevated basal levels of ROS, treatment with oxidative stressors did not induce ROS or oxidative damage in neurons. This could potentially be due to optimized endogenous antioxidant mechanisms to clear ROS in immature Hu97/18 neurons that decline with neuronal maturity, though we find this unlikely considering that cellular stress responses are impaired in HD neurons.

We did not observe cell death in either control or HD neurons following 100 μM H_2_O_2_ treatment, which is consistent with previous findings that treatment with H_2_O_2_ up to 500 μM does not increase LDH release in control or HD (ST*Hdh*^Q111^) neurons (Jin et al., 2012). However, we did find increased basal cell death in mature HD neurons. Although this, to our knowledge, has not been previously reported in HD primary forebrain neurons, viral expression of HTT fragments with expanded CAG in neurons is known to cause increased cell death over wild type HTT expression (Grima et al., 2017; Hermel et al., 2004). Additionally, several groups have reported increased caspase activation and cytochrome *c* release in HD models, suggestive of activated apoptotic cascades in HD neurons (Li et al., 2000; Hermel et al., 2004; Kim et al., 2010; Yang et al., 2010; Dickey et al., 2017). We found a trend toward increased caspase-3 activation in Hu97/18 neurons, and while this did not reach significance, we do believe this pathway could be contributing to cell death in these neurons. H_2_O_2_ has previously been shown to induce autophagy, which decreases damage in cells and protects cells from cell death at low levels, and, at higher levels, can induce autophagy-dependent apoptosis (Chen et al., 2009; Higgins et al., 2011). A previous study showed that cell death in response to H_2_O_2_ treatment is mediated through autophagic activity in cultured primary cortical neurons (Chen et al., 2009; Higgins et al., 2011). However, autophagy is impaired in HD, with HD cells exhibiting cargo recognition and loading defects that result in the accumulation of empty autophagosomes (Martinez-Vicente et al., 2010; Martin et al., 2015; Croce and Yamamoto, 2019).

We observed elevated LC3 perinuclear localization, which is indicative of increased autophagy (Lee et al., 2011), in HD neurons. Whether this vesicle formation led to increased autophagy was not directly tested in our studies, but our results are consistent with previous reports of increased LC3 expression in HD neurons (Lee et al., 2015; Ehrnhoefer et al., 2018).

In addition to basal differences between immature and mature neurons, we also found that progerin treatment effectively induced aging-related protein changes in HD mouse primary forebrain neurons, HD patient iPSC-derived neurons, and the brains of HD mice. NMDAR1 is a subunit of NMDA receptors and dynamin 1 helps facilitate endocytosis in neurons; both proteins have been shown to decrease with aging (Gazzaley et al., 1996), which is consistent with our observations in neurons treated with progerin. p16^INK4A^ is a cyclin-dependent kinase inhibitor involved in the regulation of the cell cycle which is a robust marker of aging (Ressler et al., 2006). We observed an increase in striatal p16^INK4A^ levels with progerin treatment, further suggesting that exogenous progerin induces aging-like phenotypes in neurons and brains. Moreover, irrespective of genotype, dendritic complexity, and dendrite length were both reduced by progerin treatment, which is observed in the biological aging of neurons (Dickstein et al., 2007, 2013). We also measured lamin B1, as lamin B1 is the primary nuclear lamin protein in neurons (Jung et al., 2013), and its loss is associated with cellular senescence (Freund et al., 2012). Interestingly, although progerin is a truncated form of lamin A, it also decreased levels of lamin B1 in neurons. Lamin A is not highly expressed in neurons, which is hypothesized to be the reason why HGPS does not have brain involvement (Jung et al., 2013). Our results indicate, however, that exogenous progerin expression can disrupt endogenous lamin proteins, resulting in a similar breakdown of the nuclear lamina and age-related cellular changes that are observed in cells most affected in HGPS. This can be seen through the nuclear blebbing that was observed in virtually all progerin expressing cells. While the loss of nuclear shape and integrity can be indicative of apoptosis (Daniel and DeCoster, 2004; Eidet et al., 2014), we observed blebbing in control neurons that did not have appreciable cell death or caspase-3 activation, indicating that nuclear blebbing is more likely a sign of cellular senescence in our model systems. Surprisingly, the expression of progerin in neurons also induced mtHTT oligomerization. Accumulation and aggregation of mtHTT are not seen in primary neurons from HD mice or in iPSC-derived neurons from HD patients. When age-related changes were induced with progerin, however, EM48 positive oligomers formed, suggesting that aging neurons can modulate the folding and aggregation state of mtHTT proteins irrespective of the passage of time or chronological age of cells. This is consistent with a previous report of EM48 positive mtHTT aggregates in striatal-like neurons directly converted from HD patient fibroblasts, which maintain fibroblast epigenetics, including markers of age (Victor et al., 2018). Considering the very low HTT expression in skin cells compared to neurons, these cells would have undergone a relatively short passage of time in the context of abundant mtHTT expression, only the time since conversion. Thus, mtHTT aggregation in directly converted MSNs is also not likely related to the passage of time or chronological age.

Progerin treatment also increased DNA damage and sensitized HD neurons to oxidative stress. Most cases of HD are adult-onset; however, most *in vitro* models of HD are embryonic-like, and even *in vivo* models are young at the phenotypic onset. While several models of HD display phenotypes such as increased basal and stress-induced DNA damage compared to controls (Goula et al., 2009; Chiu et al., 2015), progerin treatment enhances these phenotypes in a treatment-dependent, and possibly age-dependent manner. The evidence here, along with previous work showing progerin-induced aging can uncover phenotypes in iPSC-derived neurons from Parkinson’s disease patients (Miller et al., 2013), provides a rationale for using progerin-induced aging as a paradigm to enhance phenotypes in HD and other age-dependent neurodegenerative diseases. This will be especially important for HD, as neuronal and mouse models harboring the most common pathogenic (adult-onset) CAG repeat lengths do not display spontaneous neurodegeneration or many robust HD-like phenotypes (Hodgson et al., 1999; The HD iPSC Consortium, 2012).

**Figure 8:**
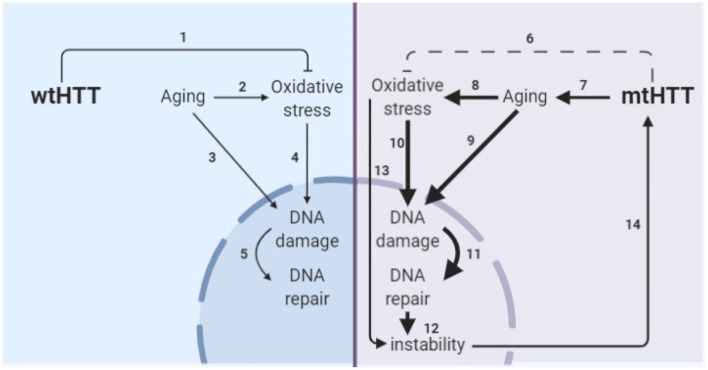
Model of the interplay of aging and oxidative stress in HD pathogenesis. **(1)** wtHTT is a stress response protein that can form Huntingtin stress bodies in the cytoplasm (Nath et al., 2015), can act as a ROS and endoplasmic reticulum (ER) stress sensor (Atwal et al., 2007; DiGiovanni et al., 2016), and aid in the base excision repair pathway in the nucleus in response to DNA damage (Maiuri et al., 2017). **(2)** Aging increases oxidative stress in cells (Beckman and Ames, 1998; Andriollo-Sanchez et al., 2005). **(3)** Aging increases DNA damage (Maynard et al., 2015). **(4)** Oxidative stress increases oxidative damage (Barzilai and Yamamoto, 2004). **(5)** DNA damage increases DNA repair particularly the base-excision repair pathway (Maynard et al., 2015; Prasad et al., 2015; Cadet et al., 2017). **(6)** mtHTT does not perform its stress response functions in the cell as well (Nath et al., 2015; Maiuri et al., 2017). Additionally, **(7–11)** accelerated epigenetic aging has been found in age-matched HD brains (Horvath et al., 2016) and age-related nuclear pore complex deficiencies have been found in HD (Grima et al., 2017), which collectively drive greater age-related oxidative stress and DNA damage and greater subsequent base excision DNA repair. **(12)** Altering the rate of base-excision repair can increase somatic instability of the CAG tract (Kovtun et al., 2007; Goula et al., 2009; Budworth et al., 2015). **(13)** Oxidative stress can cause somatic instability of the CAG tract (Jonson et al., 2013), possibly through the DNA damage/DNA repair pathway. **(14)** Somatic instability of the CAG tract is a driver of mtHTT toxicity and may be necessary for certain tissues for onset and progression of HD (Swami et al., 2009; Budworth et al., 2015; Flower et al., 2019).

The obstacle of modeling HD in developmental neurons has been addressed by another group through direct conversion of HD patient fibroblasts to striatal-like neurons (Victor et al., 2018). Directly converted neurons maintain the donor fibroblast epigenetic characteristics, including those of age and, like progerin-aged neurons, display HD-like phenotypes not seen in developmental HD or control neurons. The consistent results between these aged human neuron models; directly converted neurons with the epigenetics of skin cells and iPSC-derived neurons after induction of age-related changes by progerin treatment, suggests that this is a true feature of HD neurons rather than a model system artifact. Additionally, the consistent elevation of DNA damage selectively in HD neurons across the three model systems employed in this study provides strong evidence that age-related changes in stress and damage responses may be primary drivers of HD pathogenesis. This is particularly important because DNA damage may drive somatic expansion in HD neurons, which, in turn, may drive HD onset and progression (Wheeler et al., 2003; Dragileva et al., 2009; Swami et al., 2009), as shown in our proposed model of HD pathogenesis (Figure 8).

It should be noted that the DNA damage increase found in HD models has not been conclusively linked to aging (Goula et al., 2009), thus, further studies on this relationship are warranted with models of HD that possess an aging component. Additionally, future work should investigate directly if aging can modulate somatic instability and expansion in HD models. These findings have implications for potential modifiers of HD onset and/or progression within the aging process and suggest that anti-aging therapies and modulation of aging processes may be beneficial in the treatment or prevention of HD.

## Data Availability Statement

The datasets generated for this study are available on request to the corresponding author.

## Ethics Statement

The animal study was reviewed and approved by The Animal Care Committee of the University of British Columbia and the Institute Animal Care and Use Committee of the University of Central Florida.

## Author Contributions

AS conceived the study. EM, NC, VM, MH, and AS provided supervision and mentorship. EM, RJ, NC, SM, MS, CT, NP, YX, VM, and AS designed and performed experiments. EM, RJ, NC, MS, VM, and AS performed data analysis and interpretation. EM wrote the manuscript. RJ, NC, MS, VM, and AS assisted with writing and revision of the manuscript. All authors contributed to the article and approved the submitted version.

## Conflict of Interest

The authors declare that the research was conducted in the absence of any commercial or financial relationships that could be construed as a potential conflict of interest.
